# Vulvar Fibroadenoma: A Rare Differential Diagnosis of a Vulvar Mass

**DOI:** 10.1002/ccr3.73534

**Published:** 2026-09-11

**Authors:** Fatemeh Nili, Aysan Nozheh, Maryam Norouzi, Abolfazl Gilani

**Affiliations:** ^1^ Department of Pathology, Cancer Institute, Imam Khomeini Hospital Complex Tehran University of Medical Sciences Tehran Iran; ^2^ Faculty of Medicine Iran University of Medical Sciences Tehran Iran

**Keywords:** ectopic mammary tissue, endometrial cancer, labia majora, vulvar fibroadenoma

## Abstract

Vulvar fibroadenoma is a rare benign tumor arising from mammary‐type tissue or mammary‐like anogenital glands. A 46‐year‐old Asian woman presented with a slowly enlarging painful labial mass and a history of gynecological disorders. Excision confirmed fibroadenoma, highlighting its inclusion in the differential diagnosis of vulvar masses.

## Key Clinical Message

Vulvar fibroadenoma, though rare, should be considered in the differential diagnosis of persistent vulvar masses. Early histopathological evaluation and complete surgical excision are essential for accurate diagnosis, symptom relief, and excellent prognosis with minimal risk of recurrence.

## 1 Introduction

Mammary‐type tissue of the vulva is a rare entity first described in 1872. It was traditionally considered ectopic breast tissue resulting from incomplete regression of the embryonic milk line. Similar to normal breast tissue, it is hormonally responsive and can develop benign and malignant lesions. More recent evidence suggests these lesions arise from mammary‐like anogenital glands, normal components of the vulva that share structural and functional characteristics with breast tissue. Consequently, vulvar fibroadenoma is thought to originate predominantly from these specialized glands rather than ectopic mammary tissue. Although fibroadenoma is common in the breast, its occurrence in the vulva is extremely rare. We report a case of vulvar fibroadenoma in a woman with a history of gynecological disorders to highlight its inclusion in the differential diagnosis of vulvar masses [1, 2, 3].

## 2 Case Presentation

A 46‐year‐old Asian woman presented with a gradually enlarging brown‐colored mass on the left labia majora measuring 4 × 3 × 2.5 cm. The lesion had been present for 2 years, initially resembling acne before becoming larger, firmer, and painful. She denied discharge or pruritus but reported a 5‐kg weight loss.

Her medical history included cervical polyp, endometrial atrophy, and uterine myoma, all treated with hysterectomy. Her mother had undergone hysterectomy for endometrial cancer. She had no history of breast disease or significant comorbidities. She had one vaginal delivery at 36 weeks approximately 20 years earlier and had breastfed her child. She denied smoking, alcohol consumption, illicit drug use, and high‐risk sexual behavior.

**FIGURE 1:**
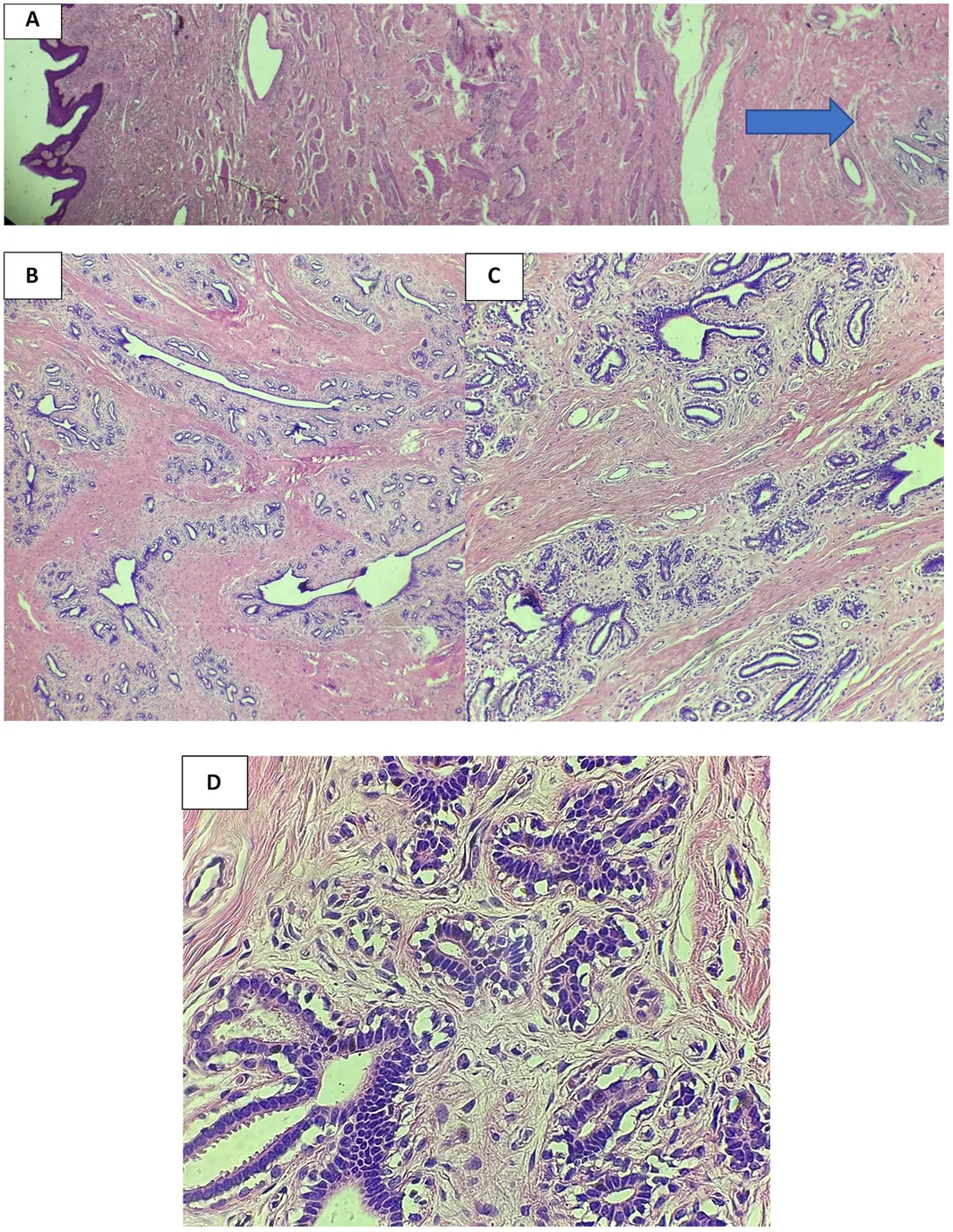
Histological sections stained with hematoxylin and eosin (H&E). (A) Whole‐slide scan of vulvar skin with the underlying lesion. (B) Lesion at ×40 magnification. (C) Lesion at ×100. (D) Lesion at ×400.

Differential diagnoses included Bartholin gland cyst, epidermal inclusion cyst, hidradenitis suppurativa, and vulvar neoplasm. The lesion was completely excised under local anesthesia. Histopathological examination demonstrated a well‐circumscribed benign biphasic fibroepithelial neoplasm composed of glandular structures lined by epithelial and myoepithelial cells within a hypocellular fibrous stroma, confirming vulvar fibroadenoma (Figure 1). The postoperative course was uneventful, with no recurrence during follow‐up.

## 3 Discussion

Vulvar fibroadenoma is a rare benign fibroepithelial tumor resembling breast fibroadenoma. Because of its rarity and nonspecific presentation, it is frequently mistaken for more common vulvar lesions. Our patient presented with a relatively large painful mass, whereas most reported cases describe smaller, asymptomatic lesions. Histopathological examination remains the gold standard for diagnosis, demonstrating the characteristic biphasic epithelial and stromal proliferation. Complete surgical excision is curative in most cases and is associated with an excellent prognosis and a very low recurrence rate. Although no established association exists between vulvar fibroadenoma and gynecological disorders, documenting such cases may improve understanding of this uncommon lesion. Vulvar fibroadenoma should be considered in the differential diagnosis of persistent vulvar masses, and definitive diagnosis relies on histopathological evaluation after complete surgical excision [1, 2, 3].

## Author Contributions

**Aysan Nozheh:** investigation, writing – original draft, visualization. **Maryam Norouzi:** writing – review and editing, resources. **Abolfazl Gilani:** conceptualization, validation, investigation, project administration, writing – original draft, writing – review and editing. **Fatemeh Nili:** conceptualization, supervision, writing – review and editing.

## Funding

The authors have nothing to report.

## Ethics Statement

The authors have nothing to report.

## Consent

Written informed consent was obtained from the patient for publication of this case and accompanying images.

## Conflicts of Interest

The authors declare no conflicts of interest.

## Data Availability

The authors have nothing to report.
